# *Lactobacillus rhamnosus* GR-1 Ameliorates *Escherichia coli*-Induced Activation of NLRP3 and NLRC4 Inflammasomes With Differential Requirement for ASC

**DOI:** 10.3389/fmicb.2018.01661

**Published:** 2018-07-24

**Authors:** Qiong Wu, Yao-Hong Zhu, Jin Xu, Xiao Liu, Cong Duan, Mei-Jun Wang, Jiu-Feng Wang

**Affiliations:** Department of Veterinary Clinical Sciences, College of Veterinary Medicine, China Agricultural University, Beijing, China

**Keywords:** bovine mammary epithelial cell, *Lactobacillus rhamnosus*, *Escherichia coli*, inflammasome, ASC

## Abstract

*Escherichia coli* is a common cause of mastitis in dairy cows. The adaptor protein apoptosis-associated speck-like protein containing a caspase recruitment domain (ASC) synergizes with caspase-1 to regulate inflammasome activation during pathogen infection. Here, the *ASC* gene was knocked out in bovine mammary epithelial (MAC-T) cells using clustered, regularly interspaced, short palindromic repeat (CRISPR)/CRISPR-associated (Cas)-9 technology. MAC-T cells were pre-incubated with and without *Lactobacillus rhamnosus* GR-1 and then exposed to *E. coli*. Western blot analysis demonstrated increased expression of NLRP3 and NLRC4 following *E. coli* infection, but this increase was attenuated by pre-incubation with *L. rhamnosus* GR-1, regardless of *ASC* knockout. Western blot and immunofluorescence analyses revealed that pre-incubation with *L. rhamnosus* GR-1 decreased *E. coli-*induced caspase-1 activation at 6 h after *E. coli* infection, as also observed in *ASC*-knockout MAC-T cells. The *E. coli-*induced increase in *caspase-4* mRNA expression was inhibited by pre-incubation with *L. rhamnosus* GR-1. *ASC* knockout diminished, but did not completely prevent, increased production of IL-1β and IL-18 and cell pyroptosis associated with *E. coli* infection, whereas pre-incubation with *L. rhamnosus* GR-1 inhibited this increase. Our data indicate that *L. rhamnosus* GR-1 suppresses activation of ASC-dependent NLRP3 and NLRC4 inflammasomes and production of downstream IL-lβ and IL-18 during *E. coli* infection. *L. rhamnosus* GR-1 also inhibited *E. coli*-induced cell pyroptosis, in part through attenuation of NLRC4 and non-canonical caspase-4 activation independently of ASC.

## Introduction

*Escherichia coli* is a frequent cause of bovine mastitis and a leading cause of clinical mastitis in bovine (Shaheen et al., 2015). The NLR family member pyrin domain-containing protein 3 (NLRP3) inflammasome is considered a suitable target for new alternatives to antibiotics to treat bovine mastitis (Thacker et al., 2012). Our previous study showed that probiotic *Lactobacillus rhamnosus* GR-1 ameliorates *E. coli*-induced inflammatory damage via attenuation of apoptosis-associated speck-like protein containing a caspase recruitment domain (ASC)-independent NLRP3 inflammasome activation in primary bovine mammary epithelial cells (PBMCs) (Wu et al., 2016). Therefore, *L. rhamnosus* GR-1 represents a potentially promising therapeutic agent targeting inflammasome activity in *E. coli*-associated bovine mastitis.

Binding of lipopolysaccharide (LPS) from gram-negative bacteria to toll-like receptor (TLR) 4 increases cellular expression of NLRP3 protein through nuclear factor-κB (NF-κB) signaling, leading to rapidly NLRP3 activation (Afonina et al., 2017). Upon activation, NLRP3 nucleates the adaptor protein ASC through interaction with the pyrin domain (PYD). Pro-caspase-1 is subsequently autoproteolytically processed through CARD–CARD (caspase recruitment domain) interactions in the NLRP3/ASC complex scaffold and cleaves precursors of the proinflammatory interleukin (IL)-1 family into their bioactive forms, IL-1β and IL-18. We found that *L. rhamnosus* GR-1 reduces *E. coli-*induced caspase-1 activation and production of IL-1β and IL-18. However, in contrast to increases in the expression of NLRP3 and caspase-1, expression of the adaptor protein ASC is decreased in PBMCs infected with *E. coli*, even in cells pretreated with *L. rhamnosus* GR-1 (Wu et al., 2016).

In contrast to the multiple stimuli that activate NLRP3, NLRC4 is activated by flagellin and the rod protein EscI of the *E. coli* type III secretion system (T3SS) apparatus (Miao et al., 2010). NLRC4 contains a CARD motif, through which it directly oligomerizes with caspase-1 independent of ASC; this complex activates caspase-1 without autoproteolysis, triggering pyroptosis, an inflammatory form of cell death (Broz et al., 2010b). However, ASC greatly enhances the efficiency of NLRC4-mediated maturation of IL-1β and IL-18 by inducing caspase-1 autoproteolysis (Lamkanfi and Dixit, 2014). NLRC4-dependent production of IL-1β is induced by pathogenic *Salmonella* or *Pseudomonas* but not commensal *Lactobacillus plantarum*, indicating that the NLRC4 inflammasome specifically discriminates pathogens and probiotic bacteria (Franchi et al., 2012). However, the contributions of the NLRC4 inflammasome to inflammatory responses that control *E. coli* infections are less clear in relation to *L. rhamnosus* GR-1.

NLRP3 and NLRC4 inflammasomes play a crucial role in potentiating the host antimicrobial response (Guo et al., 2015). Studies using ASC-deficient cells from *ASC^-/-^* mice demonstrated the dual role of ASC in bridging NLRP3 and NLRC4 inflammasomes and caspase-1 via PYD and CARD and regulating the result of inflammasome activation (Broz et al., 2010a; Gueya et al., 2014). ASC-dependent inflammasome activation results in the production of proinflammatory IL-1 family cytokines, whereas ASC-independent inflammasome activation induces cell pyroptosis. Given the significant potential of IL-1 family cytokines to cause detrimental inflammation and pyroptosis to control the spread of intracellular pathogens (Jorgensen et al., 2016; Lannitti et al., 2016), the role of ASC in regulating inflammasome activity during *E. coli* infection must be examined in detail to determine and how *L. rhamnosus* GR-1 regulates the immune response to prevent *E. coli*-associated bovine mastitis.

In the present study, we knocked out the *ASC* gene in bovine mammary epithelial (MAC-T) cells using the RNA-guided clustered regularly interspaced short palindrome repeats (CRISPR)-CRISPR-associated nuclease 9 (Cas9) system. We hypothesized that during *E. coli* infection, the activity of NLRP3 and NLRC4 inflammasomes is differentially regulated by *L. rhamnosus* GR-1, inducing maturation of IL-1β and IL-18 or cell pyroptosis, depending on ASC. We provide evidence that *L. rhamnosus* GR-1 suppresses *E. coli*-induced ASC-dependent activation of NLRP3 and NLRC4 inflammasomes and thus decreases production of IL-lβ and IL-18 during *E. coli* infection. In addition, *L. rhamnosus* GR-1 suppresses *E. coli*-induced cell pyroptosis, in part through attenuation of NLRC4 inflammasome and non-canonical caspase-4 activation, independent of ASC.

## Materials and Methods

### Biosecurity Statement

All bacterial strains were treated in strict accordance with the *Regulations on Biological Safety Management of Pathogen Microbiology Laboratory* (000014349/2004-00195) from the State Council of the People’s Republic of China. The *E. coli* CVCC1450 was subjected to all necessary safety procedures to avoid pathogen transmission and infection.

### Construction of CRISPR/Cas9 System Expression Vector

Three guide RNAs (ASC-sgRNA1, ASC-sgRNA 2, and ASC-sgRNA 3) were designed to target the exon 1 regions of the bovine ASC gene (**Table 1**). A pair of oligos for each targeting site was annealed and ligated into the *Bbs*I site of pCRISPR-sg5, which was kindly provided by Professor Sen Wu (China Agricultural University, Beijing, China), to generate pCRISPR-sg5-ASC-sgRNA1, pCRISPR-sg5-ASC-sgRNA2, and pCRISPR-sg5-ASC-sgRNA3 plasmids. All plasmids were confirmed by sequencing (Sangon Biotech, Shanghai, China).

**Table 1:** Sequences of three guide RNAs designed to target the exon 1 region of the bovine *ASC* gene and primers for PCR amplification.

Gene product^a^	Primer		Accession number
		
	Direction^b^	Sequence (5′–3′)	
ASC-sgRNA1	F	CACCGCGATGCCATC CTGGATGCGC	NM_174730.2
	R	AAACGCGCATCCAGGAT GGCATCGC	
ASC-sgRNA2	F	CACCGCTTTCAGTGCC GCTGCGGGA	NM_174730.2
	R	AAACTCCCGCAGCG GCACTGAAAGC	
ASC-sgRNA3	F	CACCGCAAGCTCGT CAGCTACTATC	NM_174730.2
	R	AAACGATAGTAGCTG ACGAGCTTGC	
ASC	F	CCAGGTTCCTGATTTG GCTAGCTA	NM_174730.2
	R	GAAGTCTCGGTCCGGAG GCCAAGG	


*^*a*^ASC, apoptosis-associated speck-like protein containing a caspase-recruitment domain; sgRNA = Cas9/single guide RNA (sgRNA). ^*b*^F, forward; R, reverse.*

### Cell Culture and Transfection

MAC-T cells transferred with the SV40 T antigen (Huynh et al., 1991) was a gift from Dr. Ying Yu (China Agricultural University). MAC-T cells were cultured in Dulbecco’s Modified Eagle medium/Ham’s F-12 medium (1:1) supplemented with 10% heat-inactivated fetal calf serum, 100 U/mL of penicillin, and 1 g/mL of streptomycin (Invitrogen, Carlsbad, CA, United States) at 37°C in an atmosphere of 5% CO_2_ and 95% air at 95% relative humidity.

Plasmid DNA for cell transfection was prepared using an Omega Endo-free Plasmid Mini Kit II (Omega Bio-Tek Inc., Doraville, GA, United States). MAC-T cells (1 × 10^6^) were electroporated with 1.5 μg of pCRISPR-W9 plasmid, 1.5 μg of pCRISPR-sg5-ASC-sgRNA plasmid, and 1 μg of pCAG-PBase plasmid using the T-020 program of an Amaxa electroporator (Lonza, Allendale, NJ, United States), in which pCRISPR-W9 encoded Cas9 nuclease and pCRISPR-sg5-ASC-sgRNA encoded ASC-sgRNA. After electroporation, 300 cells were plated in a 10-cm dish using growth medium containing 350 μg/ml of selectable marker G418 (Sigma-Aldrich, St. Louis, MO, United States). After 10 days, individual clones were picked, and clonal cell populations were expanded. Before experiments, MAC-T cells were electroporated with pmaxGFP^TM^ (Lonza) encoding green fluorescent protein to determine transfection efficiency using the T-020 and W-001 programs. MAC-T cells were chosen for CRISPR-Cas9 inactivation experiments due to their good transfection efficiency.

### Sequencing and Protein Analysis of the Gene Target Site

Genomic DNA samples were extracted using a TIANamp Genomic DNA Kit (Tiangen, Beijing, China) according to the manufacturer’s instructions, and 50 ng of DNA template was used to amplify the 630-bp fragment encompassing the gene inactivation locus in 25 μl of PCR buffer (Takara, Shiga, Japan) using primer pairs listed in **Table 1**. The resulting PCR products were purified and subsequently sequenced to identify deletions. In addition, clonal cell population whole-cell extracts were analyzed by Western blotting.

### Immunocytochemistry

The epithelial origin of MAC-T cells was tested by staining for cytokeratin 18. MAC-T cells (6 × 10^4^ cells/well) were seeded into a 24-well culture plate with glass coverslips. After 24 h, cells were washed three times with phosphate-buffered saline (PBS) and fixed with 4% paraformaldehyde for 15 min on ice. The cells were then permeabilized with 0.2% (v/v) Triton X-100 (Sigma-Aldrich) and blocked with 1% bovine serum albumin. Subsequently, cells were incubated with mouse anti-cytokeratin-18 primary monoclonal antibody at a dilution of 1:200 (Ab668; Abcam, Cambridge, United Kingdom) for 45 min at 4°C, following by incubation with secondary antibody, goat anti-mouse fluorescein isothiocyanate (FITC)-conjugated IgG (F4143; Sigma-Aldrich). Cell nuclei were stained using 4′,6′-diamidino-2-phenylindole (DAPI; Sigma-Aldrich). Coverslips were imaged on an FV1000 confocal laser scanning biological microscope (Olympus, Tokyo, Japan).

### Bacterial Strains and Growth Conditions

*Lactobacillus rhamnosus* GR-1 ATCC 55826 was purchased from the American Type Culture Collection (Manassas, VA, United States) and grown in De Man, Rogosa, and Sharpe (MRS) broth (Oxoid, Hampshire, United Kingdom) for 24 h at 37°C under microaerophilic conditions. After overnight incubation, *L. rhamnosus* GR-1 was subcultured at a dilution of 1:100 in fresh MRS broth for approximately 8 h until reaching mid-log phase [optical density (OD) at 600 nm (OD_600_) of 0.5] for all experiments.

*Escherichia coli* CVCC1450 (serotype O111:K58) was purchased from the China Institute of Veterinary Drug Center (Beijing, China) and grown in Luria–Bertani (LB) broth (Oxoid). After overnight incubation at 37°C with vigorous shaking, bacteria were diluted 1:100 in fresh LB and grown for approximately 3 h until reaching mid-log phase (OD_600_ of 0.5).

### Adhesion Assay

Wild-type (WT) and *ASC^-/-^* MAC-T cells (3 × 10^5^ cells/well) were seeded onto a six-well transwell collagen-coated polytetrafluoroethylene (PTFE) filter. Confluent cell monolayers were pretreated with *L. rhamnosus* GR-1 (3 × 10^7^ CFU) for 3 h, and then were washed three times with PBS and exposed to *E. coli* (3 × 10^7^ CFU). At 1.5, 3, and 6 h after *E. coli* challenge, the cell monolayers were washed four times with PBS to remove non-adherent bacteria and treated with 0.05% trypsin for 10 min at 37°C. Cells were harvested by centrifugation for 10 min at 4000 *g* and lysed using 100 μl of 0.2% Triton X-100 (Sigma-Aldrich) in sterile water. The populations of *E. coli* and *L. rhamnosus* GR-1 were determined on LB and MRS agar plates, respectively. The adhesion rate of *E. coli* was defined as the adhered *E. coli* population on the cells pretreated with *L. rhamnosus* GR-1 relative to the adhered *E. coli* population in the adhesion assay of *E. coli* infection alone.

### Immunofluorescence

Confluent monolayers of WT and *ASC^-/-^* MAC-T cells (6 × 10^4^ cells/well) grown on glass coverslips in a 24-well flat-bottom culture plate were treated under four different conditions, as follows: (i) medium alone (CONT); (ii) *E. coli* alone (6 × 10^6^ CFU) at a multiplicity of infection (MOI) of 100:1 (ECOL); (iii) incubation with *L. rhamnosus* GR-1 (6 × 10^6^ CFU) at a MOI of 100:1 for 3 h (LRGR); or (iv) pre-incubation with *L. rhamnosus* GR-1 (6 × 10^6^ CFU) for 3 h prior to addition of *E*. *coli* (LRGR + ECOL). At 6 h after *E. coli* infection, the cells were washed, fixed with 4% paraformaldehyde for 15 min on ice, permeabilized with 0.2% (v/v) Triton X-100 (Sigma-Aldrich), and blocked with 1% bovine serum albumin. Subsequently, the following primary monoclonal antibodies were used: mouse anti-cytokeratin-18 (Ab668, 1:200 dilution; Abcam), rabbit anti-ASC (10500-1-AP, 1:500 dilution; Proteintech Group, Chicago, IL, United States), and mouse anti-caspase-1 (22915-1-AP, 1:500 dilution; Proteintech Group). The cells were incubated with the primary antibody for 45 min at 4°C, followed by incubation with goat anti-rabbit Cy-3 (AP307F, 1:200 dilution; Sigma-Aldrich) or FITC-conjugated IgG (F-0382, 1:40 dilution; Sigma-Aldrich) as the secondary antibody. Cell nuclei were stained with DAPI. The coverslips and slides were visualized and photographed under an FV1000 confocal laser scanning biological microscope (Olympus).

### Western Blotting

WT and *ASC^-/-^* MAC-T cells (6 × 10^4^ cells/well) were seeded onto a six-well transwell collagen-coated PTFE filter and treated with *E. coli* or *L. rhamnosus* GR-1 at a MOI of 100:1, as described above. Cells were also simultaneously treated with lactate at a concentration of 0.6 g/L (equivalent to 7 mM) and *E. coli* at a MOI of 100:1. At 1.5, 3, and 6 h after *E. coli* infection, cells were extracted using Radio-Immunoprecipitation Assay buffer (Sigma-Aldrich), as previously described (Wu et al., 2016). The primary antibodies were as follows: rabbit anti-NLRP3 (19771-1-AP, 1:200 dilution; Proteintech Group), rabbit anti-NLRC4 (12421, 1:1,000 dilution; Cell Signaling Technologies Inc., Danvers, MA, United States), mouse anti-caspase-1 (sc56036) (14F468, 1:500 dilution; Santa Cruz Biotechnology, Dallas, TX, United States), rabbit anti-cleaved caspase-4 (Gln81) (GTX86890, 1:250 dilution; GeneTex, Inc., San Antonio, TX, United States), and mouse anti-glyceraldehyde-3-phosphate dehydrogenase (GAPDH, 60004-1-Ig, 1:500 dilution; Proteintech Group). Horseradish peroxidase-conjugated AffiniPure goat anti-mouse IgG (SA00001-1, 1:5,000 dilution; Proteintech Group) or goat anti-rabbit IgG (SA00001-2, 1:5,000 dilution; Proteintech Group) were used as secondary antibodies. The detection of NLRP3 and caspase-1 proteins was performed in the same gel. After NLRP3 and caspase-1 proteins were visualized, blots were then stripped using Restore Western Blot Stripping Buffer (Solarbio, Beijing, China), and re-probed with the desired antibodies for GAPDH. The OD of each band was quantified by densitometric analysis using Quantity One software (Bio-Rad Laboratories, Richmond, CA, United States). Results are presented as the ratio of the NLRP3, NLRC4, caspase p10 subunit or cleaved caspase-4 band intensity to the GAPDH band intensity.

### Lactate Dehydrogenase (LDH) Assay

The death of WT or *ASC^-/-^* MAC-T cells under the different conditions was assessed using the CytoTox 96 Non-Radioactive Cytotoxicity Assay (Promega, Madison, WI, United States) according to the manufacturer’s instructions. The assay measures the release of LDH into the supernatant, calculated as the percentage of total LDH content as determined from cell lysates (100%). LDH released by uninfected cells was used as a maximum-lysis control. The percentage of LDH released was calculated using the following equation: [(LDH infected–LDH uninfected)/(LDH total lysis–LDH uninfected)] × 100.

### Enzyme-Linked Immunosorbent Assay (ELISA)

The concentrations of IL-1β and IL-18 in cell-free supernatants of WT or *ASC^-/-^* cells were determined at 1.5, 3, and 6 h after *E. coli* infection using commercially available ELISA kits specific for bovine IL-1β (DG90995Q) and bovine IL-18 (DG91524Q; Beijing Dongge Biotechnology Co., Beijing, China).

### Quantification of Lactate Content

Cell culture supernatants were collected. Lactate content in the supernatants were quantified using the enzymatic kit K-DLATE (Megazyme, Bray, Ireland) that allows the measurement of both D-lactate and L-lactate.

### Statistical Analysis

Statistical analysis was performed using the SAS statistical software package, version 9.1 (SAS Institute Inc., Cary, NC, United States). With regard to small sample sizes, normal distribution and homogeneity of variance were assumed using the UNIVARIATE (Shapiro–Wilk test) and HOVTEST procedures. Natural logarithm transformation was performed prior to analysis for IL-1β and IL-18 data to yield a normal distribution. Statistical significance of differences was tested using ANOVA procedures, following Tukey’s honestly significant difference *post hoc* test. Data of adhesion assay were compared by an unpaired two-tailed Student’s *t*-test. Data were visualized using GraphPad Prism5 software (GraphPad Software Inc., San Diego, CA, United States). Data from adhesion assay are presented as the mean ± standard deviation (SD) and data from Western blotting, LDH, ELISA and lactate quantification assays are presented as the mean ± standard error of the mean (SEM). Results are representative of three independent experiments, each performed in triplicate. *P*-values: ^∗^*P* < 0.05; ^∗∗^*P* < 0.01; ^∗∗∗^*P* < 0.001.

## Results

### CRISPR/Cas9 Mediates Knockout of ASC in MAC-T Cells

**FIGURE 1:**
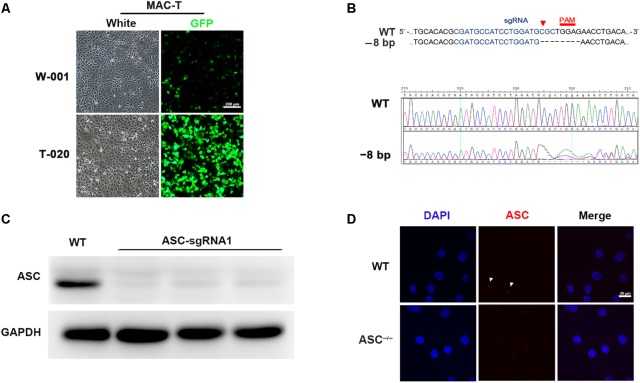
CRISPR/Cas9 system-mediated knockout of the *ASC* gene in MAC-T cells. **(A)** To determine transfection efficiency, MAC-T cells were electroporated with pmaxGFP encoding green fluorescent protein using the programs W-001 and T-020, respectively. **(B)** Schematic illustrating Cas9 inactivation of the bovine *ASC* locus. The 20-bp guide RNA target sequence is shown in blue, and the protospacer-adjacent motif (PAM) is shown in red. An 8-bp deletion was detected (Upper). Representative Sanger sequencing results of target regions of *ASC* (frameshift indels; Lower). **(C)** Analysis of ASC protein in WT and *ASC^-/-^* cells transfected with Cas-9 and ASC guide RNA expression vector by Western blotting. **(D)** Immunofluorescence staining of ASC (red) in WT and *ASC^-/-^* cells at 6 h after *Escherichia coli* challenge. DAPI was used for nuclear staining (blue). Representative confocal immunofluorescence images show staining of ASC. Scale bar, 20 μm. Data are representative of three independent experiments.

To demonstrate the role of ASC in *L. rhamnosus* GR-1 modulation of inflammasome activation during *E. coli* infection, we attempted knockout of the *ASC* gene in MAC-T cells using the CRISPR/Cas9 system. Upon immunocytochemistry analysis, MAC-T cells showed intense positive staining for epithelial cell-specific cytokeratin-18 in the cytoplasmic meshwork of cytokeratin fibrils (**Supplementary Figure S1**). Compared with program W-001, after electroporation with program T-020, MAC-T cells exhibited higher transfection efficiency (**Figure 1A**). Thus, the *ASC* gene knockout experiment was subsequently performed in MAC-T cells using program T-020. Among the three designed sgRNA sequences, the specific 20-nucleotide sgRNA1 sequence targeted the exon 1 regions of the *ASC* gene and directed Cas9 nuclease to precisely introduce a DNA double-strand break in front of a protospacer adjacent motif (PAM). After cloning and sequencing of the DNA fragment, an 8-bp deletion was observed (**Figure 1B**). Western blot analysis did not show expression of ASC protein in sgRNA1 sequence-targeted MAC-T cells (**Figure 1C**). Furthermore, *E. coli* infection triggered assembly of ASC specks in WT cells, whereas pre-incubation with *L. rhamnosus* GR-1 attenuated *E. coli-*induced ASC speck assembly. No ASC specks were observed in *ASC^-/-^* cells, regardless of *E. coli* infection (**Figure 1D**). These results demonstrated that knockout of the *ASC* gene in MAC-T cells was successful.

### Pre-incubation With *L. rhamnosus* GR-1 Reduces the Adhesion of *E. coli* to MAC-T Cell Monolayers

**FIGURE 2:**
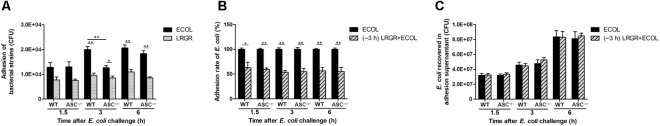
Pre-incubation with *Lactobacillus rhamnosus* GR-1 reduced the adhesion of *E. coli* to MAC-T cell monolayers. An adhesion assay was performed. Cells and supernatants were collected. The number of adherent *E. coli* and *L. rhamnosus* GR-1 was determined **(A)**. The adhesion rate of *E. coli* was defined as the adhered *E. coli* population on the cells pretreated with *L. rhamnosus* GR-1 relative to the adhered *E. coli* population in the adhesion assay of *E. coli* infection alone **(B)**. The number of *E. coli* recovered was determined in adhesion assay supernatants **(C)**. Data are presented as the mean ± SD of three independent experiments. Asterisks indicate the significances among different treatments in the same cell type. Pound signs indicate the significance among WT and *ASC^-/-^* MAC-T cells infected with *E. coli* alone at 3 h. ^∗^*P* < 0.05, ^∗∗^*P* < 0.01, ^##^*P* < 0.01.

*Escherichia coli* or *L. rhamnosus* GR-1 exhibited similar adhesion capacity in WT and *ASC^-/-^* MAC-T cells. The number of adherent *E. coli* was about 1.29 × 10^4^ ± 0.67 × 10^2^ CFU (means ± standard deviation) at 1.5 h after *E. coli* infection, and increased to 1.95 × 10^4^ ± 1.17 × 10^3^ CFU at 6 h. At 3 h after *E. coli* challenge, the number of adherent *E. coli* in *ASC^-/-^* cells was lower (*P* = 0.007) than in WT MAC-T cells (**Figure 2A**). *L. rhamnosus* GR-1 had a lower adhesion capacity and the number of adherent *L. rhamnosus* GR-1 was about 8.75 × 10^3^ ± 1.56 × 10^3^ CFU, regardless of infection time. Pre-incubation with *L. rhamnosus* GR-1 resulted in a reduction in the *E. coli* adhesion rate to 57% of that observed in MAC-T cells infected with *E. coli* alone (**Figure 2B**). No differences were observed in the number of *E. coli* recovered from the supernatant fraction among two groups (**Figure 2C**).

### Pre-incubation With *L. rhamnosus* GR-1 Attenuates *E. coli*-Induced NLRP3 Expression and Increases Lactate Content in the Supernatants

**FIGURE 3:**
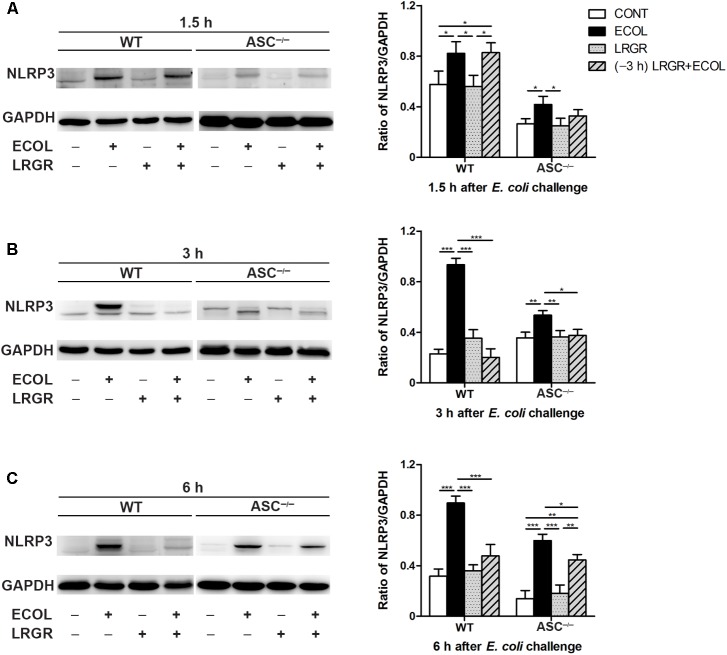
Pre-incubation with *L. rhamnosus* GR-1 attenuated *E. coli-*induced NLRP3 expression. Western blot detection of NLRP3 in WT and *ASC^-/-^* cells collected from the indicated cell cultures at 1.5 h **(A)**, 3 h **(B)**, and 6 h **(C)** after *E. coli* challenge. Representative panels showing expression of NLRP3 protein (Left). Results are presented as the ratio of NLRP3 band intensity to that of GAPDH (Right). Data are presented as the mean ± SEM of three independent experiments. ^∗^*P* < 0.05, ^∗∗^*P* < 0.01, ^∗∗∗^*P* < 0.001.

Compared with untreated control WT cells, Western blot analysis showed an increase in NLRP3 protein expression at 1.5, 3, and 6 h after *E. coli* challenge in cells only infected with *E. coli*, but not in cells incubated with *L. rhamnosus* GR-1 alone (*P* = 0.046, *P* < 0.001, and *P* < 0.001, respectively; **Figures 3A–C**). In contrast, WT cells pre-incubated with *L. rhamnosus* GR-1 had a lower expression of NLRP3 protein than did WT cells only infected with *E. coli* at 3 and 6 h (*P* < 0.001 for both).

Compared with WT cells, *ASC^-/-^* MAC-T cells exhibited a similar differential response to *E. coli* challenge and *L. rhamnosus* GR-1 incubation. Compared with untreated control *ASC^-/-^* cells, at 1.5, 3, and 6 h after *E. coli* challenge, NLRP3 protein expression was elevated in *ASC^-/-^* cells only infected with *E. coli* (*P* = 0.036, *P* = 0.005, and *P* < 0.001, respectively; **Figures 3A–C**) but not in *ASC^-/-^* cells pre-incubated with *L. rhamnosus* GR-1.

The lactate content (D-lactate plus L-lactate) in the supernatants was quantified. Compared with untreated control cells, the lactate content was increased in the supernatants from both WT and *ASC^-/-^* cells incubated with *L. rhamnosus* GR-1 alone or pre-incubated with *L. rhamnosus* GR-1, but not the cells only infected with *E. coli* at 1.5, 3, and 6 h after infection, regardless of *ASC* knockout (*P* < 0.05; **Supplementary Figure S2A**). Western blot analysis showed that compared with untreated control cells, at 6 h after *E. coli* challenge, NLRP3 protein expression was elevated in WT or *ASC^-/-^* cells infected with *E. coli*, but not in cells only treated with lactate alone (*P* < 0.05; **Supplementary Figure S2B**). Lactate addition did not attenuate *E. coli*-induced increase in NLRP3 protein expression in either WT or *ASC^-/-^* cells.

### Effect of *L. rhamnosus* GR-1 on NLRC4 Activation During *E. coli* Infection

**FIGURE 4:**
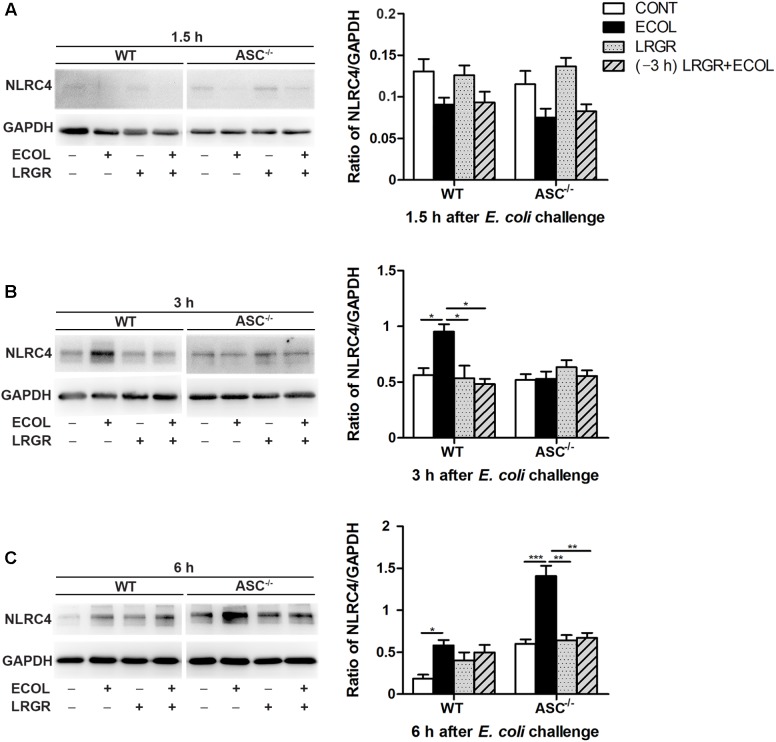
Western blot detection of NLRC4 protein. Representative panels showing expression of NLRP4 protein in WT and *ASC^-/-^* cells collected from the indicated cultures at 1.5 h **(A)**, 3 h **(B)**, and 6 h **(C)** after *E. coli* challenge (Left). Results are presented as the ratio of NLRC4 band intensity to that of GAPDH (Right). Data are presented as the mean ± SEM of three independent experiments. ^∗^*P* < 0.05, ^∗∗^*P* < 0.01, ^∗∗∗^*P* < 0.001.

Compared with untreated control cells, expression of NLRC4 protein was elevated at 3 h after *E. coli* infection in WT cells only infected with *E. coli* (*P* = 0.027; **Figure 4B**) but not in WT cells incubated with *L. rhamnosus* GR-1 alone or pre-incubated with *L. rhamnosus* GR-1. Compared with WT cells only infected with *E. coli*, expression of NLRC4 protein at 3 h was decreased in WT cells incubated with *L. rhamnosus* GR-1 alone and pre-incubated with *L. rhamnosus* GR-1 (*P* = 0.019 and *P* = 0.001, respectively). No differences were observed at 3 h in *ASC^-/-^* cells, regardless of treatment.

Compared with untreated control cells, *E. coli* challenge led to increased expression of NLRC4 protein at 6 h after *E. coli* challenge in WT cells (*P* = 0.019) and *ASC^-/-^* cells (*P* < 0.001; **Figure 4C**). Expression of NLRC4 protein was lower in *ASC^-/-^* cells incubated with *L. rhamnosus* GR-1 alone and pre-incubated with *L. rhamnosus* GR-1 (*P* = 0.001 for both) than in *ASC^-/-^* cells only infected with *E. coli*. No changes were observed at 1.5 h after *E. coli* infection in WT cells or *ASC^-/-^* cells, regardless of treatment (**Figure 4A**).

### Pre-incubation With *L. rhamnosus* GR-1 Attenuates *E. coli*-Induced Caspase-1 Maturation

**FIGURE 5:**
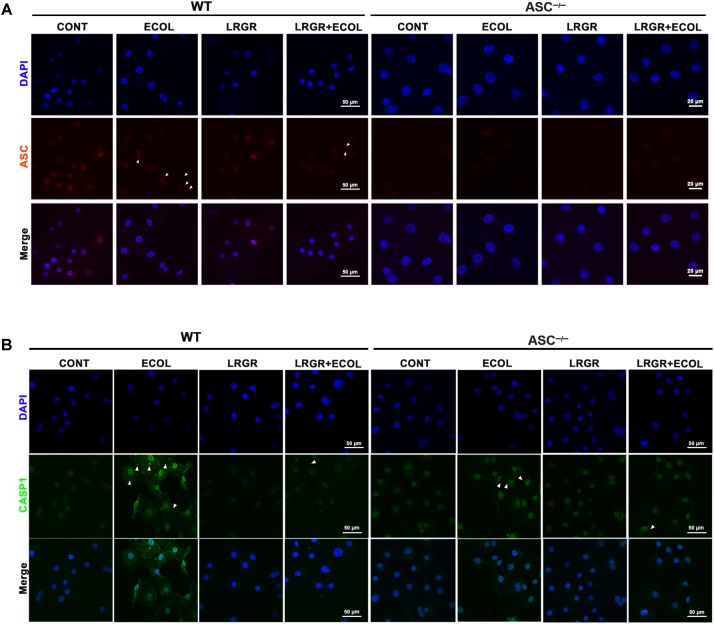
Immunofluorescence staining for ASC and caspase-1. Immunofluorescence staining for ASC **(A)** and caspase-1 **(B)** in WT and *ASC^-/-^* cells collected from the indicated cell cultures at 6 h after *E. coli* challenge. Cells were immunostained for ASC (red) and caspase-1 (green). DAPI (blue) was used to localize nuclei. Arrows mark specks. Scale bars, immunofluorescence staining for ASC in WT cells and for caspase-1 in WT and *ASC^-/-^* cells: 50 μm, and immunofluorescence staining for ASC in *ASC^-/-^* cells: 20 μm. Data are representative of three independent experiments.

Immunofluorescence staining showed that compared with untreated control cells, *E. coli* challenge triggered assembly of ASC specks in WT cells at 6 h after *E. coli* challenge, and this was attenuated in WT cells pre-incubated with *L. rhamnosus* GR-1 (**Figure 5A**). No ASC specks were observed in *ASC^-/-^* cells, regardless of treatment. Compared with untreated control cells, bright foci indicative of increased caspase-1 expression was observed in WT cells only infected with *E. coli* at 6 h after challenge; this increase was attenuated by incubation with *L. rhamnosus* GR-1 (**Figure 5B**). Compared with WT cells, ASC deletion attenuated, but did not abolish, caspase-1 staining in response to *E. coli* infection and pre-incubation with *L. rhamnosus* GR-1. A punctate staining pattern for caspase-1 was observed in *ASC^-/-^* cells only infected with *E. coli*, and pre-incubation with *L. rhamnosus* GR-1 inhibited the *E. coli-*induced punctate caspase-1 staining pattern. Compared with untreated control cells, incubation with *L. rhamnosus* GR-1 only did not result in increase in caspase-1 activation either in WT or *ASC^-/-^* cells (**Figure 5B**).

**FIGURE 6:**
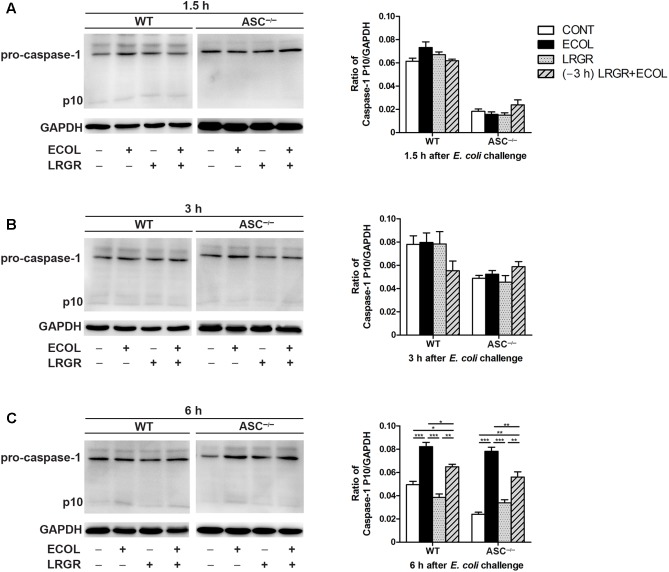
Pre-incubation with *L. rhamnosus* GR-1 attenuated *E. coli*-induced activation of caspase-1. Western blot detection of caspase-1 in WT and *ASC^-/-^* cells collected from the indicated cell cultures at 1.5 h **(A)**, 3 h **(B)**, and 6 h **(C)** after *E. coli* challenge. Representative panels showing expression of caspase-1 (Left). Results are presented as the ratio of caspase-1 p10 subunit band intensity to that of GAPDH (Right). The detection of NLRP3 and caspase-1 proteins was performed in the same gel. Caspase-1 blots shared the same GAPDH blots with NLRP3 protein. Data are presented as the mean ± SEM of three independent experiments. ^∗^*P* < 0.05, ^∗∗^*P* < 0.01, ^∗∗∗^*P* < 0.001.

Compared with untreated control cells, increased maturation of procaspase-1 into its catalytic 10-kDa subunit was observed at 6 h after *E. coli* challenge in WT and *ASC^-/-^* cells only infected with *E. coli* (*P* < 0.001 for both; **Figure 6C**). However, maturation of caspase-1 declined in WT and *ASC^-/-^* cells pre-incubated with *L. rhamnosus* GR-1 (*P* = 0.014 and *P* = 0.006, respectively) or incubated with *L. rhamnosus* GR-1 alone (*P* < 0.001 for both), compared with WT cells infected with *E. coli* only. No differences were observed in caspase-1 maturation at 1.5 and 3 h after *E. coli* infection in WT cells or *ASC^-/-^* cells, regardless of treatment (**Figures 6A,B**).

### Pre-incubation With *L. rhamnosus* GR-1 Attenuates *E. coli*-Induced Caspase-4 Activation

**FIGURE 7:**
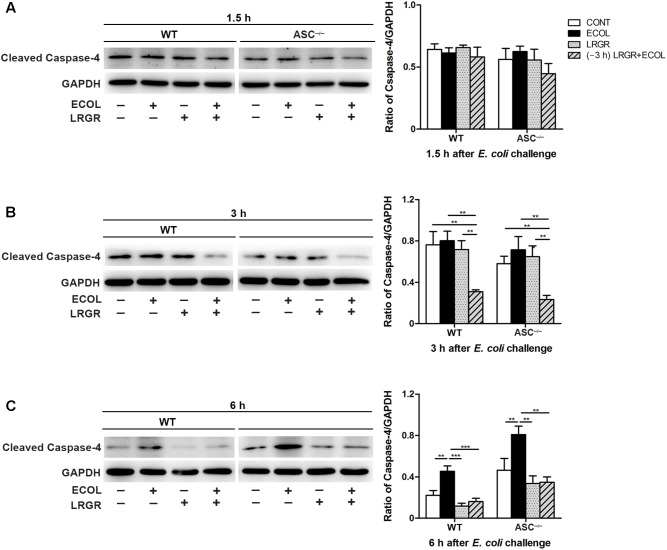
Pre-incubation with *L. rhamnosus* GR-1 attenuated *E. coli*-induced activation of caspase-4. Western blot detection of cleaved caspase-4 in WT and *ASC^-/-^* cells collected from the indicated cell cultures at 1.5 h **(A)**, 3 h **(B)**, and 6 h **(C)** after *E. coli* challenge. Representative panels showing expression of cleaved caspase-4 (Left). Results are presented as the ratio of cleaved caspase-4 band intensity to that of GAPDH (Right). Data are presented as the mean ± SEM of three independent experiments. ^∗∗^*P* < 0.01, ^∗∗∗^*P* < 0.001.

At 3 h after *E. coli* infection, expression of cleaved caspase-4 (26 kDa) in WT and *ASC^-/-^* cells pre-incubated with *L. rhamnosus* GR-1 was lower than in untreated control cells and cells only infected with *E. coli* (**Figure 7B**). Infection with *E. coli* resulted in increased expression of cleaved caspase-4 in WT and *ASC^-/-^* cells (*P* = 0.001 and *P* = 0.004, respectively; **Figure 7C**) at 6 h. However, expression of caspase-4 decreased in WT and *ASC^-/-^* cells pre-incubated with *L. rhamnosus* GR-1, compared with cells only infected with *E. coli* (*P* < 0.001 and *P* = 0.001, respectively). No differences were observed in the expression of cleaved caspase-4 at 1.5 h after *E. coli* infection in WT or *ASC^-/-^* cells, regardless of treatment (**Figure 7A**).

### Pre-incubation With *L. rhamnosus* GR-1 Attenuates *E. coli*-Induced Production of IL-1β and IL-18 and Cell Pyroptosis

**FIGURE 8:**
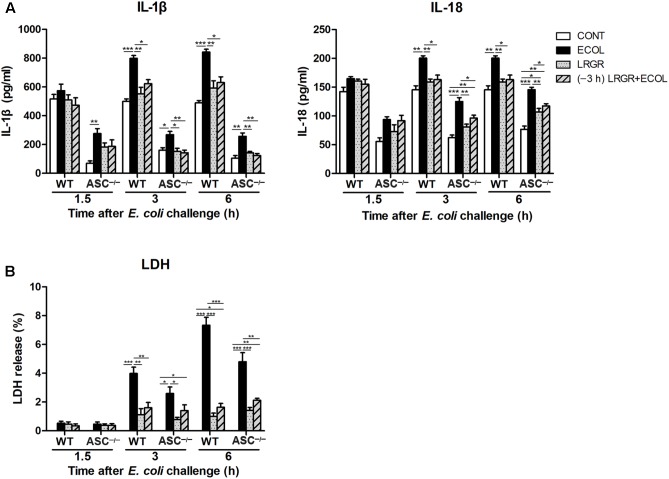
Pre-incubation with *L. rhamnosus* GR-1 attenuated *E. coli*-induced production of IL-1β and IL-18 and cell pyroptosis. Secretion of IL-1β and IL-18 **(A)** into the indicated cell culture supernatants at 1.5, 3, and 6 h after *E. coli* challenge as determined by ELISA. Cell pyroptosis was determined by measurement of LDH release **(B)**. Data are presented as the mean ± SEM of three independent experiments. ^∗^*P* < 0.05, ^∗∗^*P* < 0.01, ^∗∗∗^*P* < 0.001.

Compared with untreated control cells, IL-1β production was increased at 3 and 6 h after *E. coli* infection in WT cells only infected with *E. coli* (*P* < 0.001 for both; **Figure 8A**). IL-1β production was lower at 3 and 6 h both in WT cells incubated with *L. rhamnosus* GR-1 alone (*P* = 0.002 and *P* = 0.004, respectively) and cells pre-incubated with *L. rhamnosus* GR-1 (*P* = 0.013 and *P* = 0.011, respectively) than in WT cells only infected with *E. coli*. Compared with WT cells, *ASC* deletion led to a decrease in production of IL-1β in *ASC^-/-^* cells in response to different treatments. Challenge with *E. coli* resulted in elevated production of IL-1β at 1.5, 3, and 6 h after *E. coli* infection in *ASC^-/-^* cells only infected with *E. coli* (*P* = 0.009, *P* = 0.021, and *P* = 0.001, respectively; **Figure 8A**) compared with untreated control cells; however, the *E. coli-*induced increase in IL-1β production was attenuated at 3 and 6 h by incubation with *L. rhamnosus* GR-1 alone (*P* = 0.014 and *P* = 0.007, respectively) and pre-incubation with *L. rhamnosus* GR-1 (*P* = 0.009 and *P* = 0.003, respectively).

IL-18 exhibited similar differential production as IL-1β. Compared with untreated control cells, production of IL-18 was increased at 3 and 6 h after *E. coli* challenge both in WT (*P* = 0.001 for both) and *ASC^-/-^* (*P* < 0.001 for both) cells only infected with *E. coli* (**Figure 8A**). However, incubation with *L. rhamnosus* GR-1 alone and pre-incubation with *L. rhamnosus* GR-1 attenuated the *E. coli-*induced elevation in the concentration of IL-18 at 3 and 6 h after *E. coli* challenge both in WT and *ASC^-/-^* cells. Compared with untreated control *ASC^-/-^* cells, the concentration of IL-18 was elevated at 3 and 6 h in *ASC^-/-^* cells pre-incubated with *L. rhamnosus* GR-1 (*P* = 0.008 and *P* = 0.002, respectively).

Cell pyroptosis was quantified by monitoring the release of LDH into the supernatants after *E. coli* challenge. Compared with untreated control cells, the percentage of pyroptotic cells at 3 and 6 h was increased in WT cells only infected with *E. coli* (*P* < 0.001 for both; **Figure 8B**) but not in WT cells incubated with *L. rhamnosus* GR-1 alone and pre-incubated with *L. rhamnosus* GR-1. Compared with WT cells, *ASC* deletion let to a similarly differential but attenuated cell pyroptosis. Compared with untreated control cells, the percentage of pyroptotic cells was increased at 3 and 6 h in *ASC^-/-^* cells only infected with *E. coli* (*P* = 0.020 and *P* < 0.001, respectively), whereas incubation with *L. rhamnosus* GR-1 alone and pre-incubation with *L. rhamnosus* GR-1 ameliorated the *E. coli-*induced increase in pyroptotic cell death at 6 h (*P* < 0.001 and *P* = 0.002, respectively). No changes were observed in WT and *ASC^-/-^* cells, regardless of treatment.

## Discussion

Bacterial adhesion to host epithelial cells is an essential step in the initiation of infection. *Lactobacillus* can reduce pathogen adhesion to epithelial cells and exert direct antimicrobial activity due to accumulation of antimicrobial substances (Gudina et al., 2015). We found that *L. rhamnosus* GR-1 did not directly kill *E. coli*, but did decrease the level of *E. coli* adhesion to 57% of that observed in MAC-T cells infected with *E. coli* alone. We previously revealed that live and ultraviolet-irradiated *L. rhamnosus* GR-1 rather than culture supernatant of *L. rhamnosus* GR-1 and medium acidified with lactate lead to a decrease in the *E. coli* adhesion rate in bovine mammary epithelial cells (Wu et al., 2016). The reduced *E. coli* adhesion level mediated by *L. rhamnosus* GR-1 may be attributed to steric hindrance due to competition for attachment sites (Ardita et al., 2014; Tytgat et al., 2016).

NLRP3 is activated by a wide variety of stimuli, including pore-forming toxins, extracellular adenosine triphosphate, RNA-DNA hybrid molecules, and pathogens (Jo et al., 2016). We found that *E. coli* infection also increased the expression of NLRP3 protein from 1.5 to 6 h, but *L. rhamnosus* GR-1 pretreatment inhibited this increase. NLRP3 must be primed before activation. *Escherichia coli* LPS binds to TLR4 to induce expression of NLRP3 protein via NF-κB signaling. Bacterial mRNA from viable *E. coli* cells that have been phagocytosed enters the cytosolic compartment, resulting in assembly of the NLRP3 inflammasome (Sander et al., 2011). Intake of *L. plantarum* CECT 7315/7316 downregulates expression of *Nlrp3* in the ileum of rats (Vilahur et al., 2015). However, the NLRP3 inflammasome is also activated by *L. rhamnosus* GG and LC705 originating from dairy sources (Miettinen et al., 2012).

In a mouse immune hepatitis model, lactate treatment was shown to attenuate hepatic and pancreatic injury by negatively regulating TLR4-mediated activation of the NLRP3 inflammasome and production of IL-1β through arrestin β2 and G-protein-coupled receptor 81 (Hoque et al., 2014). In the present study, there was a higher lactate content in the supernatants of cells incubated with *L. rhamnosus* GR-1. However, additional lactate treatment did not attenuate the *E. coli*-induced increase in expression of NLRP3. This indicates that the elevated lactate content in the supernatant is a secondary effect of *L. rhamnosus* GR-1 treatment and cannot account for attenuation of *E. coli*-induced activation of NLRP3. Previously, we have shown that *L. rhamnosus* GR-1 attenuates *E. coli*-induced TLR4 expression in bovine mammary epithelial cells (Wu et al., 2016). This may contribute to attenuating the priming step and subsequent activation of NLRP3 through TLR4-mediated NF-κB signaling (Bauernfeind et al., 2009). The mitogen-activated protein kinase (MAPK) subfamilies, c-Jun N-terminal kinase (JNK) and extracellular regulated protein kinase (ERK) are essential for NLRP3 inflammasome activation and addition of JNK1/2 inhibitor SP600125 or upstream MAPK/ERK kinase inhibitor PD98059 of ERK inhibits the LPS-induced increase in NLRP3 protein expression (Liao et al., 2013). Culture medium of *L. rhamnosus* GR-1 inhibits LPS-induced JNK activation in macrophages or monocytic THP-1 cells (Kim et al., 2006). Histamine derived from *Lactobacillus reuteri* 6475 inhibits activation of ERK in THP-1 cells (Thomas et al., 2012). It is possible that *L. rhamnosus* GR-1 attenuates *E. coli*-induced NLRP3 activation through reducing the adhesion of *E. coli* to MAC-T cells and subsequently negatively regulating the functional synergy between NF-κB and JNK/ERK MAPK pathways mediated by some uncertain soluble factors. Further studies are required to determine active components derived from *L. rhamnosus* GR-1 and elucidate possible mechanisms underlying the antagonistic effects of *L. rhamnosus* GR-1 on NLRP3 activation during *E. coli* infection.

NLRP3 contains only a PYD, which engages the PYD of ASC, leaving the CARD of ASC to interact with the CARD-containing region of pro-caspase-1. Caspase-1 is thought to be activated by a proximity-induced dimerization and autoproteolytic process in the NLRP3/ASC complex platform (Shi, 2004). Active caspase-1 cleaves pro-IL-lβ and pro-IL-18 into mature IL-lβ and IL-18, which are essential for coordination of immune responses to pathogen infection through allograft neutrophil sequestration, mononuclear phagocyte recruitment, and T-cell activation (Samuel Weigt et al., 2017). In the present study, *L. rhamnosus* GR-1 attenuated *E. coli-*induced caspase-1 autoproteolysis and elevated production of mature IL-lβ and IL-18 at 6 h after challenge. Another study also showed that the NLRP3 inflammasome pathway plays a critical role in the host immune response to pathogen infection (Dikshit et al., 2018). However, inappropriate activation of the NLRP3 inflammasome is linked not only to local inflammation but also several autoimmune inflammatory disorders in humans (Seo et al., 2015). Indeed, activation of the NLRP3 inflammasome amplifies inflammation and promotes pathogen infection via a process involving triggering of T helper 2-biased adaptive immune responses (Gurung et al., 2015) or secretion of secondary danger-associated molecular pattern molecules (Bui et al., 2016). Our data suggest that *L. rhamnosus* GR-1 prevents *E. coli*-induced inflammation by suppressing activation of ASC-dependent NLRP3 inflammasomes.

In mice, non-canonical caspase-11 was identified as a key regulator of NLRP3 inflammasome-associated caspase-1 activation in response to *E. coli* infection. Caspase-11 is activated via NLRP3-independent mechanisms, but it is essential for NLRP3-dependent and ASC-dependent caspase-1 processing and IL-1β maturation in response to *E. coli* infection (Kayagaki et al., 2011). In addition, binding of LPS to human caspase-4 or murine caspase-11 via the CARD directly induces cell pyroptosis, independently of NLRP3 and ASC (Shi et al., 2014). A recent study revealed that outer-membrane-vesicle-mediated cytoplasmic delivery of extracellular *E. coli* LPS activates murine caspase-11 to induce pyroptosis and IL-1β maturation (Vanaja et al., 2016). Bovine caspase-4 is a homolog of human caspase-4 and mouse caspase-11 and plays a role in the processing of IL-1β and IL-18 precursors (Koenig et al., 2001; Martinon and Tschopp, 2004). In the present study, caspase-4 was activated by *E. coli* at 6 h; however, *L. rhamnosus* GR-1 pretreatment attenuated this activation.

Interestingly, there was a lower number of adherent *E. coli* in *ASC^-/-^* MAC-T cells at 3 h after *E. coli* challenge compared with WT MAC-T cells. It must be noted that ASC deficiency reduced, but did not abolish, caspase-1 processing, IL-1β and IL-18 maturation, and cell pyroptosis during *E. coli* infection. Decreased *E. coli* adhesion may delay the expression of NLRC4 receptor and the downstream activation of caspase-1, maturation of proinflammatory cytokines and cell pyroptosis in *ASC^-/-^* MAC-T cells. Caspase-1 activation by *E. coli* requires NLRP3 and ASC, but caspase-11 processing and cell pyroptosis do not (Kayagaki et al., 2011). A previous study reported weaker oligomerization of both ASC and caspase-1 in macrophages infected with *E. coli* compared with the canonical NLRP3 inflammasome activator nigericin, but with comparable production of IL-1β (Rathinam et al., 2012). Caspase-11 interacts with caspase-1 in infected cells, forming a heterodimeric complex (Kayagaki et al., 2011). These data suggest that caspase-4/-11 amplify caspase-1 activation independently of ASC by enabling caspase-1 autoprocessing through heterodimerization. Indeed, in the present study, caspase-4 activation was enhanced in *ASC^-/-^* cells compared with WT cells, which could have compensated for the loss of caspase-1 activation due to ASC-dependent NLRP3 inflammasome activation. Our findings indicate that *E. coli* infection activates caspase-4, subsequently resulting in cell pyroptosis and maturation of IL-1β and IL-18 via an NLRP3 inflammasome-dependent and ASC-independent pathway. *Lactobacillus rhamnosus* GR-1 suppresses ASC-dependent NLRP3 inflammasome activation and ASC-independent caspase-1 processing by inhibiting caspase-4 activation, thereby attenuating cell pyroptosis and cytokine production and thus preventing establishment of *E. coli* infection.

In contrast to NLRP3 activation in response to diverse stimuli, upon *E. coli* infection, NLRC4 responds to bacterial rod protein of the T3SS apparatus and flagellin (Zhao et al., 2011). We found that *L. rhamnosus* GR-1 inhibited *E. coli*-induced NLRC4 expression, as was also observed in *ASC^-/-^* cells. NLRC4 contains a CARD motif through which it directly interacts with caspase-1 to induce pyroptosis, independently of ASC. This NLRC4-dependent/ASC-independent cell death pathway proceeds in the absence of caspase-1 autoproteolysis. Interestingly, we observed weaker staining for caspase-1 in *ASC^-/-^* cells. Caspase-1 autoproteolysis is often used as an indicator of caspase-1 activation. However, it was also reported that uncleaved caspase-1 is enzymatically active in *ASC^-/-^* cells and can induce pyroptosis. In contrast to the formation of a single large ASC/caspase-1 focus for efficient IL-lβ and IL-18 processing, pro-caspase-1 could be recruited to NLRC4, with which it forms a smaller complex that induces pyroptosis (Broz et al., 2010b). Although NLRC4 contains a CARD, ASC amplifies NLRC4 inflammasome activity because ASC is essential for NLRC4-induced caspase-1 autoprocessing and maturation of IL-lβ and IL-18 (Brubaker et al., 2015). Our data suggest that *L. rhamnosus* GR-1 inhibits *E. coli*-induced cell pyroptosis via suppression of ASC-independent NLRC4 inflammasome activation. During *E. coli* infection in the present study, *L. rhamnosus* GR-1 decreased the secretion of IL-lβ and IL-18, in part due to suppression of ASC-dependent NLRC4 inflammasome activation.

This MAC-T cell model of *E. coli* and *L. rhamnosus* GR-1 co-incubation presents an *in vitro* framework for assessing bovine mammary immune response to pathogen infection, and evaluating the efficiency of *Lactobacillus*-based intervention in preventing bovine mastitis. The results require further confirmation in other cell lines and *in vivo* studies. However, several concerns need to be addressed before the clinical application of *Lactobacillus* in bovine mastitis. Oral ingestion of probiotics promotes mucosal immune response to pathogen infection in the gut. The mechanism underlying how the immunomodulatory effect extends to the mammary glands remains unclear. The means of probiotic supplementation (e.g., mixed into the feed, oral capsules or intra-mammary infection) and dose effect also needs to be studied in more details. The molecular mechanism underlying regulation of inflammasome activity by *Lactobacillus* requires further investigation. Our findings identify NLRP3 and NLRC4 inflammasomes as potential targets for bovine mastitis therapy and could strengthen the development for other inflammasome-targeted therapies in *E. coli*-associated mastitis.

## Conclusion

**FIGURE 9:**
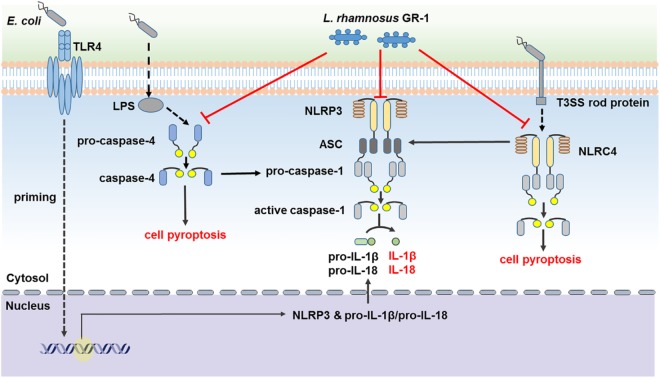
*Lactobacillus rhamnosus* GR-1 attenuated activation of ASC-dependent and ASC-independent inflammasomes during *E. coli* infection. *L. rhamnosus* GR-1 ameliorates *E. coli-*induced inflammatory damage via attenuation of both ASC-dependent and ASC-independent inflammasome activation in MAC-T cells. *L. rhamnosus* GR-1 inhibits activation of ASC-dependent NLRP3 and NLRC4 inflammasomes and production of the downstream proinflammatory cytokines IL-lβ and IL-18 during *E. coli* infection. *L. rhamnosus* GR-1 suppresses *E. coli-*induced cell pyroptosis, in part through attenuation of NLRC4 inflammasome activation, independently of ASC. In addition, *L. rhamnosus* GR-1 inhibits non-canonical caspase-4 activation, which subsequently synergizes with the NLRP3 inflammasome to attenuate caspase-1 activation and potentially inhibit caspase-1-independent cell pyroptosis and IL-lβ and IL-18 production. Full lines represent the results of the present study, and dashed lines represent the conclusions drawn in other studies.

In conclusion, our findings suggest that *L. rhamnosus* GR-1 ameliorates *E. coli-*induced inflammatory damage by attenuating both ASC-dependent and ASC-independent inflammasome activation in MAC-T cells (**Figure 9**). *L. rhamnosus* GR-1 inhibits activation of ASC-dependent NLRP3 and NLRC4 inflammasome activation and production of the downstream proinflammatory cytokines IL-lβ and IL-18 during *E. coli* infection. In addition, *L. rhamnosus* GR-1 suppresses *E. coli-*induced cell pyroptosis, in part through attenuation of NLRC4 inflammasome activation, independently of ASC. Furthermore, *L. rhamnosus* GR-1 inhibits non-canonical caspase-4 activation, which subsequently synergizes with NLRP3-/ASC-dependent caspase-1 activation to potentially inhibit ASC-independent caspase-1 activation, thus suppressing cell pyroptosis and IL-lβ and IL-18 production.

## Author Contributions

QW, Y-HZ, and J-FW conceived and designed the experiments, analyzed the data, and wrote the manuscript. QW, JX, XL, CD, and M-JW performed the experiments.

## Conflict of Interest Statement

The authors declare that the research was conducted in the absence of any commercial or financial relationships that could be construed as a potential conflict of interest.

## Supplementary Material

FIGURE S1 Immunodetection of cytokeratin-18 in MAC-T cells. Representative confocal immunocytochemistry images showing typical morphology of MAC-T cells. MAC-T cells were immunostained for cytokeratin-18 (red). DAPI (blue) was used to localize nuclei. Scale bar, 20 μm. Data are representative of three independent experiments.

FIGURE S2 The lactate content in the cell supernatants and the effect of lactate on NLRP3 expression during *E. coli* infection. Lactate content in the supernatants was determined **(A)**. Cells were also simultaneously treated with lactate (LACT) at a concentration of 0.6 g/L (equivalent to 7 mM) and *E. coli* at a MOI of 100:1. Western blot detection of NLRP3 in WT and *ASC^-/-^* cells collected from the indicated cell cultures at 6 h after *E. coli* challenge **(B)**. Representative panels showing expression of NLRP3 protein (Left). NLRP3 band intensity was determined using Quantity One software. Results are presented as the ratio of NLRP3 band intensity to that of GAPDH (Right). Data are presented as the mean ± SEM of three independent experiments. ^∗^*P* < 0.05, ^∗∗^*P* < 0.01, ^∗∗∗^*P* < 0.001.

## References

[B1] Afonina I. S. Zhong Z. Karin M. Beyaert R. (2017). Limiting inflammation-the negative regulation of NF-kappaB and the NLRP3 inflammasome. *Nat. Immunol.* 18 861–869. 10.1038/ni.3772 28722711

[B2] Ardita C. S. Mercante J. W. Kwon Y. M. Luo L. Crawford M. E. Powell D. N. (2014). Epithelial adhesion mediated by pilin SpaC is required for *Lactobacillus rhamnosus* GG-induced cellular responses. *Appl. Environ. Microbiol.* 80 5068–5077. 10.1128/AEM.01039-14 24928883 PMC4135752

[B3] Bauernfeind F. G. Horvath G. Stutz A. Alnemri E. S. MacDonald K. Speert D. (2009). NF-kB activating pattern recognition and cytokine receptors license NLRP3 inflammasome activation by regulating NLRP3 expression. *J. Immunol.* 183 787–791. 10.4049/jimmunol.0901363 19570822 PMC2824855

[B4] Broz P. Newton K. Lamkanfi M. Mariathasan S. Dixit V. M. Monack D. M. (2010a). Redundant roles for inflammasome receptors NLRP3 and NLRC4 in host defense against *Salmonella*. *J. Exp. Med.* 207 1745–1755. 10.1084/jem.20100257 20603313 PMC2916133

[B5] Broz P. von Moltke J. Jones J. W. Vance R. E. Monack D. M. (2010b). Differential requirement for caspase-1 autoproteolysis in pathogen-induced cell death and cytokine processing. *Cell Host Microbe* 8 471–483. 10.1016/j.chom.2010.11.007 21147462 PMC3016200

[B6] Brubaker S. W. Bonham K. S. Zanoni I. Kagan J. C. (2015). Innate immune pattern recognition: a cell biological perspective. *Annu. Rev. Immunol.* 33 257–290. 10.1146/annurev-immunol-032414-112240 25581309 PMC5146691

[B7] Bui F. Q. Johnson L. Roberts J. Hung S. C. Lee J. Atanasova K. R. (2016). *Fusobacterium nucleatum* infection of gingival epithelial cells leads to NLRP3 inflammasome-dependent secretion of IL-1beta and the danger signals ASC and HMGB1. *Cell Microbiol.* 18 970–981. 10.1111/cmi.12560 26687842 PMC5101013

[B8] Dikshit N. Kale S. D. Khameneh H. J. Balamuralidhar V. Tang C. Y. Kumar P. (2018). NLRP3 inflammasome pathway has a critical role in the host immunity against clinically relevant *Acinetobacter baumannii* pulmonary infection. *Mucosal Immunol.* 11 257–272. 10.1038/mi.2017.50 28612844

[B9] Franchi L. Kamada N. Nakamura Y. Burberry A. Kuffa P. Suzuki S. (2012). NLRC4-driven production of IL-1beta discriminates between pathogenic and commensal bacteria and promotes host intestinal defense. *Nat. Immunol.* 13 449–456. 10.1038/ni.2263 22484733 PMC3361590

[B10] Gudina E. J. Fernandes E. C. Teixeira J. A. Rodrigues L. R. (2015). Antimicrobial and anti-adhesive activities of cell-bound biosurfactant from *Lactobacillus agilis* CCUG31450. *RSC Adv.* 5 90960–90968. 10.1039/C5RA11659G

[B11] Gueya B. Bodnara M. Maniéa S. N. Tardivele A. Petrilli V. (2014). Caspase-1 autoproteolysis is differentially required for NLRP1b and NLRP3 inflammasome function. *Proc. Natl. Acad. Sci. U.S.A.* 111 17254–17259. 10.1073/pnas.1415756111 25404286 PMC4260594

[B12] Guo H. Callaway J. B. Ting J. P. (2015). Inflammasomes: mechanism of action, role in disease, and therapeutics. *Nat. Med.* 21 677–687. 10.1038/nm.3893 26121197 PMC4519035

[B13] Gurung P. Karki R. Vogel P. Watanabe M. Bix M. Lamkanfi M. (2015). An NLRP3 inflammasome-triggered Th2-biased adaptive immune response promotes leishmaniasis. *J. Clin. Invest.* 125 1329–1338. 10.1172/JCI79526 25689249 PMC4362229

[B14] Hoque R. Farooq A. Ghani A. Gorelick F. Mehal W. Z. (2014). Lactate reduces liver and pancreatic injury in Toll-like receptor- and inflammasome-mediated inflammation via GPR81-mediated suppression of innate immunity. *Gastroenterology* 146 1763–1774. 10.1053/j.gastro.2014.03.014 24657625 PMC4104305

[B15] Huynh H. T. Robitaille G. Turner J. D. (1991). Establishment of bovine mammary epithelial cells (MAC-T): an in vitro model for bovine lactation. *Exp. Cell Res.* 197 191–199. 10.1016/0014-4827(91)90422-Q 1659986

[B16] Jo E. K. Kim J. K. Shin D. M. Sasakawa C. (2016). Molecular mechanisms regulating NLRP3 inflammasome activation. *Cell. Mol. Immunol.* 13 148–159. 10.1038/cmi.2015.95 26549800 PMC4786634

[B17] Jorgensen I. Lopez J. P. Laufer S. A. Miao E. A. (2016). IL-1beta, IL-18, and eicosanoids promote neutrophil recruitment to pore-induced intracellular traps following pyroptosis. *Eur. J. Immunol.* 46 2761–2766. 10.1002/eji.201646647 27682622 PMC5138142

[B18] Kayagaki N. Warming S. Lamkanfi M. Vande Walle L. Louie S. Dong J. (2011). Non-canonical inflammasome activation targets caspase-11. *Nature* 479 117–121. 10.1038/nature10558 22002608

[B19] Kim S. O. Sheikh H. I. Ha S. Martins A. Reid G. (2006). G-CSF-mediated inhibition of JNK is a key mechanism for *Lactobacillus rhamnosus*-induced suppression of TNF production in macrophages. *Cell. Microbiol.* 8 1958–1971. 10.1111/j.1462-5822.2006.00763.x 16889627

[B20] Koenig U. Eckhart L. Tschachler E. (2001). Evidence that caspase-13 is not a human but a bovine gene. *Biochem. Biophys. Res. Commun.* 285 1150–1154. 10.1006/bbrc.2001.5315 11478774

[B21] Lamkanfi M. Dixit V. M. (2014). Mechanisms and functions of inflammasomes. *Cell* 157 1013–1022. 10.1016/j.cell.2014.04.007 24855941

[B22] Lannitti R. G. Napolioni V. Oikonomou V. De Luca A. Galosi C. Pariano M. (2016). IL-1 receptor antagonist ameliorates inflammasome-dependent inflammation in murine and human cystic fibrosis. *Nat. Commun.* 7:10791. 10.1038/ncomms10791 26972847 PMC4793079

[B23] Liao P. C. Chao L. K. Chou J. C. Dong W. C. Lin C. N. Lin C. Y. (2013). Lipopolysaccharide/adenosine triphosphate-mediated signal transduction in the regulation of NLRP3 protein expression and caspase-1-mediated interleukin-1β secretion. *Inflamm. Res.* 62 89–96. 10.1007/s00011-012-0555-2 22986467

[B24] Martinon F. Tschopp J. (2004). Inflammatory caspases: linking an intracellular innate immune system to autoinflammatory diseases. *Cell* 117 561–574. 10.1016/j.cell.2004.05.004 15163405

[B25] Miao E. A. Mao D. P. Yudkovsky N. Bonneau R. Lorang C. G. Warren S. E. (2010). Innate immune detection of the type III secretion apparatus through the NLRC4 inflammasome. *Proc. Natl. Acad. Sci. U.S.A.* 107 3076–3080. 10.1073/pnas.0913087107 20133635 PMC2840275

[B26] Miettinen M. Pietila T. E. Kekkonen R. A. Kankainen M. Latvala S. Pirhonen J. (2012). Nonpathogenic *Lactobacillus rhamnosus* activates the inflammasome and antiviral responses in human macrophages. *Gut Microbes* 3 510–522. 10.4161/gmic.21736 22895087 PMC3495788

[B27] Rathinam V. A. Vanaja S. K. Waggoner L. Sokolovska A. Becker C. Stuart L. M. (2012). TRIF licenses caspase-11-dependent NLRP3 inflammasome activation by gram-negative bacteria. *Cell* 150 606–619. 10.1016/j.cell.2012.07.007 22819539 PMC3660860

[B28] Samuel Weigt S. Palchevskiy V. Belperio J. A. (2017). Inflammasomes and IL-1 biology in the pathogenesis of allograft dysfunction. *J. Clin. Invest.* 127 2022–2029. 10.1172/JCI93537 28569730 PMC5451233

[B29] Sander L. E. Davis M. J. Boekschoten M. V. Amsen D. Dascher C. C. Ryffel B. (2011). Detection of prokaryotic mRNA signifies microbial viability and promotes immunity. *Nature* 474 385–389. 10.1038/nature10072 21602824 PMC3289942

[B30] Seo S. U. Kamada N. Munoz-Planillo R. Kim Y. G. Kim D. Koizumi Y. (2015). Distinct commensals induce interleukin-1beta via NLRP3 inflammasome in inflammatory monocytes to promote intestinal inflammation in response to injury. *Immunity* 42 744–755. 10.1016/j.immuni.2015.03.004 25862092 PMC4408263

[B31] Shaheen M. Tantary H. A. Nabi S. U. (2015). A treatise on bovine mastitis: disease and disease economics, etiological basis, risk factors, impact on human health, therapeutic management, prevention and control strategy. *Adv. Dairy Res.* 4:150.

[B32] Shi J. Zhao Y. Wang Y. Gao W. Ding J. Li P. (2014). Inflammatory caspases are innate immune receptors for intracellular LPS. *Nature* 514 187–192. 10.1038/nature13683 25119034

[B33] Shi Y. (2004). Caspase activation: revisiting the induced proximity model. *Cell* 117 855–858. 10.1016/j.cell.2004.06.007 15210107

[B34] Thacker J. D. Balin B. J. Appelt D. M. Sassi-Gaha S. Purohit M. Rest R. F. (2012). NLRP3 inflammasome is a target for development of broad-spectrum anti-infective drugs. *Antimicrob. Agents Chemother.* 56 1921–1930. 10.1128/AAC.06372-11 22290938 PMC3318317

[B35] Thomas C. M. Hong T. Peter van Pijkeren J. Hemarajata P. Trinh D. V. Hu W. (2012). Histamine derived from probiotic *Lactobacillus reuteri* suppresses TNF via modulation of PKA and ERK signaling. *PLoS One* 7:e31951. 10.1371/journal.pone.0031951 22384111 PMC3285189

[B36] Tytgat H. L. P. Douillard F. P. Reunanen J. Rasinkangas P. Hendrickx A. P. Laine P. K. (2016). *Lactobacillus rhamnosus* GG outcompetes *Enterococcus faecium* via mucus-binding pili: evidence for a novel and heterospecific probiotic mechanism. *Appl. Environ. Microbiol.* 82 5756–5762. 10.1128/AEM.01243-16 27422834 PMC5038030

[B37] Vanaja S. K. Russo A. J. Behl B. Banerjee I. Yankova M. Deshmukh S. D. (2016). Bacterial outer membrane vesicles mediate cytosolic localization of LPS and caspase-11 activation. *Cell* 165 1106–1119. 10.1016/j.cell.2016.04.015 27156449 PMC4874922

[B38] Vilahur G. Lopez-Bernal S. Camino S. Mendieta G. Padro T. Badimon L. (2015). *Lactobacillus plantarum* CECT 7315/7316 intake modulates the acute and chronic innate inflammatory response. *Eur. J. Nutr.* 54 1161–1171. 10.1007/s00394-014-0794-9 25408198

[B39] Wu Q. Liu M. C. Yang J. Wang J. F. Zhu Y. H. (2016). *Lactobacillus rhamnosus* GR-1 ameliorates *Escherichia coli*-induced inflammation and cell damage via attenuation of ASC-independent NLRP3 inflammasome activation. *Appl. Environ. Microbiol.* 82 1173–1182. 10.1128/AEM.03044-15 26655757 PMC4751844

[B40] Zhao Y. Yang J. Shi J. Gong Y. Lu Q. Xu H. (2011). The NLRC4 inflammasome receptors for bacterial flagellin and type III secretion apparatus. *Nature* 477 596–600. 10.1038/nature10510 21918512

