# Supplementary figures and images for: Lactobacillus rhamnosus GR-1 Ameliorates Escherichia coli-Induced Activation of NLRP3 and NLRC4 Inflammasomes With Differential Requirement for ASC
Source: Front Microbiol. 2018 Jul 24;9:1661. doi: 10.3389/fmicb.2018.01661 (PMC6066506; doi:10.3389/fmicb.2018.01661)

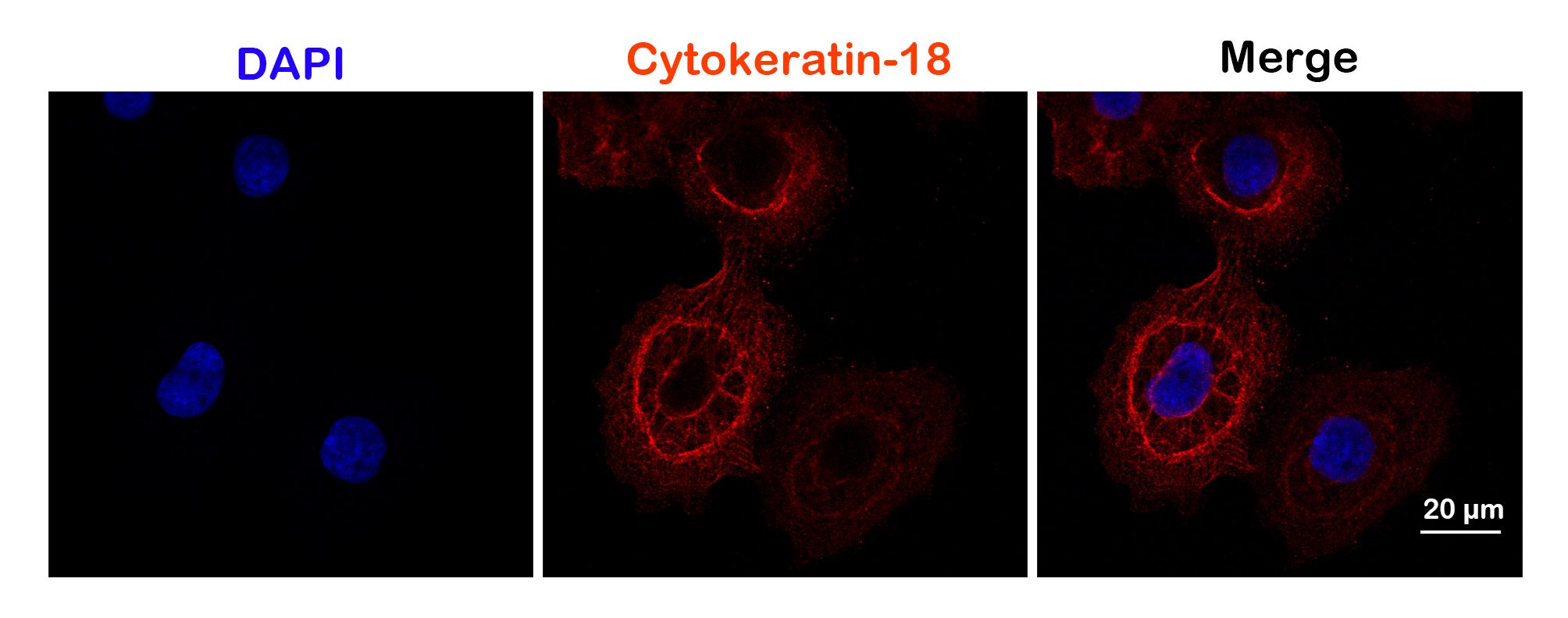

Supplement: FIGURE S1 — Immunodetection of cytokeratin-18 in MAC-T cells. Representative confocal immunocytochemistry images showing typical morphology of MAC-T cells. MAC-T cells were immunostained for cytokeratin-18 (red). DAPI (blue) was used to localize nuclei. Scale bar, 20 μm. Data are representative of three independent experiments. [file Image_1.TIF]

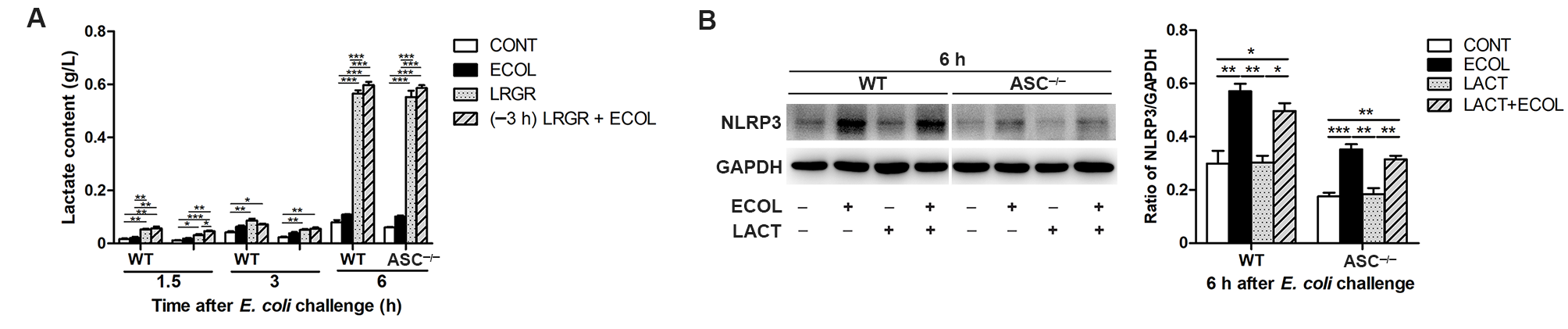

Supplement: FIGURE S2 — The lactate content in the cell supernatants and the effect of lactate on NLRP3 expression during E. coli infection. Lactate content in the supernatants was determined (A). Cells were also simultaneously treated with lactate (LACT) at a concentration of 0.6 g/L (equivalent to 7 mM) and E. coli at a MOI of 100:1. Western blot detection of NLRP3 in WT and ASC-/- cells collected from the indicated cell cultures at 6 h after E. coli challenge (B). Representative panels showing expression of NLRP3 protein (Left). NLRP3 band intensity was determined using Quantity One software. Results are presented as the ratio of NLRP3 band intensity to that of GAPDH (Right). Data are presented as the mean ± SEM of three independent experiments. ∗P < 0.05, ∗∗P < 0.01, ∗∗∗P < 0.001. [file Image_2.TIF]
